# Immunoglobulin A protease from *Sutterella wadsworthensis* modifies outcome of infection with *Campylobacter jejuni* and is associated with microbiome diversity

**DOI:** 10.1080/19490976.2025.2611543

**Published:** 2026-01-06

**Authors:** Marwan E. Majzoub, Fernando S. Santiago, Shreeya S. Raich, Prakruti Sirigeri, Isidora Simovic, Nicodemus Tedla, Nadeem O. Kaakoush

**Affiliations:** a School of Biomedical Sciences, Faculty of Medicine and Health, UNSW Sydney, Sydney, NSW, Australia; b School of Biotechnology and Biomolecular Sciences, Faculty of Science, UNSW Sydney, Sydney, NSW, Australia

**Keywords:** IgA-specific serine endopeptidases, microbiome, *Sutterella*, pathogenesis, metagenomics

## Abstract

*Sutterella wadsworthensis* is an enigmatic member of the microbiota, previously reported to be present in healthy humans yet also associated with certain gut diseases and their therapeutic outcomes. Here, we report on *S. wadsworthensis* classified to *S. wadsworthensis*_A that encodes an immunoglobulin A (IgA) protease that digests human IgA1 and IgA2 but not mouse IgA. The activity of this IgA protease could influence the trajectory of *Campylobacter jejuni* infection in human epithelial cells and phagocytosis in primary neutrophils. Comparative genomics and screening of metagenomic samples revealed that the protease shared sequence identity with an IgA protease from a bacterium that colonized other mammals and that *S. wadsworthensis* harboring IgA protease can be detected in individuals globally. Individuals positive for *S. wadsworthensis* IgA protease in China and Fiji (detection at >90% similarity) were found to have a different microbiome when compared to individuals where the protease was not detected. Phylogenetic analysis of pathogen IgA proteases along with IgA proteases from members of the microbiota suggested that there may be a unique subset of microbiota-derived IgA proteases. Our results highlight the importance of taxonomic resolution in microbiome studies and identify a subgroup of *S. wadsworthensis* that may be of potential clinical relevance.

## Background

The production of antibodies is fundamental to a properly functioning immune system. Of the five classes, immunoglobulin A (IgA) is the most abundantly produced by far, with the majority localized at mucosal sites. Unlike circulating IgA that is produced by plasma cells in bone marrow as monomer (mIgA), mucosal IgA is produced by local plasma cells as a dimer (dIgA). Humans produce two IgA subtypes, IgA1 and IgA2, the main difference being the lack of 13 amino acids from the hinge region of IgA2.^1^

IgA is best known for its capacity for immune exclusion, pathogen neutralization, and antigen excretion. In the gut, dIgA produced in the lamina propria is transported across the epithelium by poly Ig receptor and secreted to the lumen where it binds to bacteria or their products to stop their interaction with epithelial cells.^2^^,^^3^ Further, even prior to secretion, during transport through epithelial cells, IgA can bind intracellular bacteria or antigens and help shuttle them to the lumen.^4^ In the gut, IgA helps the immune system differentiate between commensals and pathogens by acting as a second signal that enables the conversion of pattern recognition receptor activation to inflammation.^1^^,^^5^

IgA plays an influential role in susceptibility to microbial infections, and its role in other diseases has been revealed from data on IgA-deficient individuals. Despite many of these individuals having sub-clinical presentation, they have a significantly increased risk (up to ~10-fold) of developing chronic inflammatory disorders such as rheumatoid arthritis, systemic lupus erythematosus, celia disease, and inflammatory bowel diseases (IBD) - both Crohn’s disease and ulcerative colitis (UC) - as well as cancer.^1^^,^^6^^,^^7^ The reasons for this are still not fully elucidated; however, it is likely that the lack of IgA-associated immunity and tissue repair play an important role.

There is a constant dialog between our gut microbiota and resident immune cells that contributes to production of substantial levels of IgA from stimulated B-cells.^8^ However, members of the microbiota differ in how they interact with IgA and in their capacity to induce its production.^9^ Some microbes possess an ability to degrade IgA through expression of IgA-specific serine endopeptidases.^10^ This activity has been commonly associated with human pathogens such as *Neisseria*, *Haemophilus* and *Streptococcus* species.^10^ IgA proteases of these bacteria cleave the hinge region of IgA1 but less so the shorter hinge in IgA2, eliminating critical functions mediated by the Fc region including Fc-receptor dependent bacterial phagocytosis. Notably, other mammals including mice produce only one type of IgA that differs from human IgA1, explaining host specificity of these pathogens and their enzymes.^11^

*Sutterella wadsworthensis* is member of the human and mouse microbiotas that has an ambiguous relationship with its host.^12^ Its presence or abundance has been associated with negative outcomes of both standard and microbial therapeutics in UC;^13-17^ yet, it has very mild pro-inflammatory capacity and no impact on monolayer integrity when cultured with intestinal epithelial cells.^18^^,^^19^ Global phenotypic, genotypic or proteomic differences among strains isolated from patients with UC and controls do not explain these findings.^19^ To investigate this further, we recently searched for putative virulence factors within *S. wadsworthensis* genomes and identified a gene encoding a putative IgA protease within a genome of a *S. wadsworthensis* strain.^12^ This gene was rare as it was not present in any of the other available *S. wadsworthensis* genomes at the time (*n* > 40).^12^ *Sutterella* spp. are readily detected on gut mucosal surfaces,^18^ where secretory IgA exerts selective pressure on members of the gut microbiota, making the possession of IgA protease activity by this mucosal bacterium potentially relevant to colonization outcomes of other bacteria.

Thus, to explore the relationship between *S. wadsworthensis* and its host further and provide insights on microbiota-derived IgA proteases, we investigated i) the ability of *S. wadsworthensis* putative IgA protease to degrade IgA and the consequences on bacterial pathogenesis; ii) how it acquired the gene encoding the protease; iii) the global prevalence of the gene encoding the protease in metagenomic data; iv) the microbiome profile of individuals positive for this protease; and v) the capacity to identify putative IgA proteases from other members of the microbiota using the novel IgA protease sequence.

## Material and methods

### Cloning, expression and purification of S. wadsworthensis IgA protease

A recognition sequence for BamHI was added before the 5’ translation start site of the *S. wadsworthensis* IgA protease coding sequence (CDS) (*Sutterella* sp. KLE1602 GenBank LTDB01000061.1, CDS = 10669-14724). The stop codon was deleted before 6x His (CAC nucleotides), a stop codon and BamHI recognition sequence were inserted at the 3’-end. This modified CDS was ligated into BamHI-digested pET-29a(+) (generated by GenScript). pET-29a(+) containing the *S. wadsworthensis* IgA protease CDS was DNA sequenced and digested with BamHI to confirm the insert as well as its orientation. The plasmid was then transformed into BL21 (DE3) competent cells (Thermo Fisher Scientific) and plated on Luria-Bertani (LB) agar plate with kanamycin (50 µg/ml, Sigma-Aldrich). A single transformant was inoculated into 50 ml LB broth with kanamycin and incubated overnight at 37 °C with shaking at 200 RPM. The overnight culture was added into 1 l LB broth with kanamycin and incubated at 37 °C for 2 h with constant shaking at 200 RPM. Isopropyl *β*-D-1-thiogalactopyranoside (1 µM IPTG, Sigma-Aldrich) was added and induction was performed by incubating the culture at 30 °C for 4 h at 200 RPM. The cell pellet was obtained by centrifugation at 5,000 RPM for 15 min and the medium was stored at −80 °C. The cell pellet was washed twice with cold 1X phosphate buffered saline (PBS) prior to resuspension with 10 ml of 1X Tris-buffered saline (TBS). Lysozyme (100 µg/ml; Sigma-Aldrich) was added to the resuspended cells and incubated at 30 °C for 30 min. After addition of Triton X-100 (1% final concentration), the lysate was sonicated (Kontes micro ultrasonic cell disrupter) on ice with settings; 4 × 30 s with output control at 5 and duty cycle at 50. DNA in the lysate was digested by incubation with DNAse I (5 U/ml; Sigma-Aldrich) for 1 h at 37 °C. Crude cell lysate was obtained by centrifugation at 13,000 RPM for 30 min at 4 °C using an Avanti J-26S high performance centrifuge (Beckman Colter) and the insoluble fraction was stored at −80 °C. The cell lysate was dialyzed against 50 mM sodium phosphate, 300 mM NaCl pH 7.5, and 10 mM imidazole (Sigma-Aldrich) prior to HisPur™ cobalt resin (Thermo Fisher Scientific) isolation and purification. His-tagged IgA protease was eluted with 50 mM sodium phosphate, 30 mM NaCl and 150 mM imidazole. The IgA protease fraction in snakeskin dialysis tubing (Thermo Fisher Scientific) was dialyzed against 50 mM Tris pH 7.5, 150 mM NaCl prior to *in vitro* digestion of human and mouse IgA substrates.

### Western blotting of digested commercial human IgA1, human IgA2 and mouse IgA

Native human IgA1 protein (Abcam #ab9102), native human IgA2 (Abcam #ab91021) and mouse IgA, kappa(S107) (Abcam #ab37322) were diluted with 50 mM Tris pH 7.5; 150 mM NaCl to a final concentration of 0.5 µg/µl. One µl of each of these IgA substrates was added to 30 µl of the *S. wadsworthensis* IgA protease or *Neisseria gonorrhoeae* IgA protease (MoBiTec cat# EP0205) or pepsin (Sigma-Aldrich cat#P6887) controls and then incubated at 37 °C for 2 h. The digestion reaction was loaded into 4−20% mini-protean TGX precast protein gels (BioRad) and electrophoresed at 100 V for 1.2 h in denaturing and reducing conditions. Proteins were transferred to Millipore Immobilon polyvinylidene difluoride membranes (Millipore) before incubation with 5% non-fat skim milk for 1 h at room temperature. Membranes were incubated overnight at 4 °C with constant gentle mixing with the appropriate antibody (mouse monoclonal antibody to human IgA1 Abcam #ab128791, mouse polyclonal to human IgA2; Abcam #ab88250, or goat polyclonal antibody to mouse IgA alpha chain-Horseradish; Abcam #ab97235) at a dilution of 1:1000 in 5% non-fat skim milk. Membranes except that of mouse IgA were washed three times with 1X PBS-0.1% Tween 20 prior to incubation with the horseradish peroxidase–linked secondary antibodies (1:5000 in 5% skim milk). Detection using ImageQuant LAS4000 (GE) was achieved with chemiluminescence (Perkin‐Elmer, Waltham, MA).

### Förster resonance energy transfer (FRET) to assess IgA protease activity

FRET human IgA1 derived peptide F2.2A with EDANS as donor and DABCL as quencher was used as a substrate to assess IgA1 protease activity. To confirm sensitivity and specificity of the assay, two modified FRET peptides were also generated by inserting two amino acids (F2.2B) or by using a peptide of 9 amino acid residues homologous to the human IgA1 hinge (F2.2C) (Mimotopes; Mulgrave VIC). All peptides were dissolved with dimethyl sulfoxide (DMSO) and further diluted to 10 μM with 50 mM Tris; 150 mM NaCl pH 7.5. 100 μl of IgA protease was mixed with 8 μl FRET peptide substrate (0.75 μM) and loaded into pre-warmed (37 °C) SpectraMax M3 plate reader (Molecular Devices; San Jose, CA). EDANS fluorescence emission (λ_ex_ = 340 nm, λ_em_ = 485 nm with automatic cut-off at 475 nm) was monitored every 15 min for 6 h.

### *Western blotting of digested* Campylobacter jejuni-specific *recombinant IgA1 and IgA2*

Recombinant monoclonal human immunoglobulin A antibodies against *C. jejuni* flagellar capping protein FliD^20^ were obtained from Humabs Biomed SA - VIR Biotechnology. These include monomeric IgA1, dimeric IgA1, monomeric IgA2 and dimeric IgA2. Digestion of these four antibodies by *S. wadsworthensis* IgA protease was confirmed by western blotting as performed for commercial IgA.

### Gentamicin protection assay

HT-29 monolayers were infected at a Multiplicity of Infection (MOI) of 100 with *C. jejuni* 81116 that was pre-incubated for 30 min with either vehicle, *C. jejuni*-specific mIgA1, *C. jejuni*-specific mIgA1 digested with *S. wadsworthensis* IgA protease, *C. jejuni*-specific dIgA1 and *C. jejuni*-specific dIgA1 digested with *S. wadsworthensis* IgA protease. The 24-well plates were then centrifuged at 1200 RPM for 5 min to promote contact. Following incubation for 6 h at 37 °C under microaerobic conditions, monolayers were washed and treated with 200 µg/ml of gentamicin (Gibco) for 1 h to kill extracellular bacteria. Monolayers were lysed in 1% Triton X-100 and plated on horse blood agar. Colony-forming units were then counted after 3 d at 37 °C. Statistical analysis on relative intracellular levels (normalized to intracellular levels without IgA) was performed in GraphPad Prism v10 using unpaired t-test following data distribution and variance testing with the Shapiro-Wilk test and F-test.

### Enrichment of peripheral blood neutrophils

Ethics approval for collection of blood from healthy donors and isolation of neutrophils was obtained from the UNSW Human Research Ethics Committee (approval number: iRECS7258). Primary human neutrophils were prepared from 20 ml of fresh peripheral blood in BD Vacutainer Citrate tubes (Becton Dickinson) by dextran precipitation of red blood cells followed by gradient centrifugation of the buffy using Ficoll/Hypaque (Amersham Pharmacia).^21^ Briefly, the peripheral blood was poured into 50 ml falcon tubes, diluted 1:1 in PBS and mixed with 10 ml of 4.5% Dextran 500 (Sigma-Aldrich) in PBS. The buffy coat and red blood sediment were then allowed to separate in a 37 °C humidified incubator with 5% CO_2_ for 30 min. The buffy coat (~15 ml) was collected and overlayed onto 20 ml of Ficoll, centrifuged at 1500 RPM for 30 min at room temperature and the pellet containing neutrophils washed with 50 ml of PBS three times and resuspended in RPMI media containing 1.25% FBS at 1 × 10^7^/ml. The enrichment of neutrophils, assessed by Differential Quick Stain III Kit (Polyscience Inc), was >85% (range 85–92%). The contaminating cells were red blood cells, few eosinophils and occasional mononuclear cells. Viability, assessed by exclusion of trypan blue (Sigma-Aldrich), was ≥90% (range 90–100%).

### Preparation of fluorescein 5(6)-isothiocyanate (FITC)-labeled bacteria

A freshly prepared *Campylobacter jejuni* 81116 pellet (1 × 10^9^ CFU/ml) in 1.5 ml microcentrifuge tube was resuspended in 1 ml of 0.5 mg/ml of FITC (Sigma-Aldrich) in 50 mM sodium carbonate and 100 mM sodium chloride, pH, 8.0. Tubes were then incubated at 37 °C in a bacterial shaker incubator at 200 RPM for 1 h and centrifuged at 14,000 RPM for 10 min at 4 °C. The bacterial pellet was washed twice using 1 ml of wash buffer containing Hank’s Buffered Salt solution + 0.25% BSA + 2 mM HEPES and centrifuged at 14,000 RPM for 10 min at 4 °C. Labeled bacteria were then resuspended in 1 ml PBS and some were opsonised with specific antibodies and the remaining kept as non-opsonised controls.

### Antibody opsonisation of FITC-labeled bacteria

50 µl aliquots of the FITC-labeled bacteria (5 × 10^7^ CFU/ml) were incubated with 20 µg/ml of intact or *S. wadsworthensis* IgA protease-digested anti-*C. jejuni* monomeric (clone X5V), or dimeric (clone C4X) IgA1 antibodies (Vir Biotechnology) for 2 h at 37 °C with intermittent agitations. The antibody opsonised bacteria were then washed twice with 1 ml of PBS by centrifugation at 1200 RPM for 5 minutes at room temperature and subsequently resuspended in 50 µl of RPMI media (Gibco) containing 1.25% FBS and 10 mM HEPES (Gibco).

### Antibody dependent cellular phagocytosis assay and IL-8 ELIZA

50 µl aliquots (5 × 10^5^ cells) of the enriched primary neutrophils were added onto 50 µl of each of the above IgA1 opsonised or non-opsonised bacteria in 1.5 ml microcentrifuge tubes in duplicates and were incubated for 5 h at 37 °C, humidified incubator with 5% CO_2_ in a final volume of 600 µl RPMI containing 1.25% FBS and 10 mM HEPES. Neutrophils without bacteria were used as controls. After the 5-h incubation, one of each duplicate was washed twice with 1 ml of cold PBS containing 0.05% NaN_3_ and 1% BSA by centrifugation at 1200 RPM, 4 °C for 5 min then, fixed in 400 µL of 2% paraformaldehyde (Sigma, USA) and transferred to 4 °C in the dark overnight until data were acquired by flow cytometer. A total of 2 × 10^4^ events were acquired using the BD LSR Fortessa X20^TM^ Flow Cytometer and the proportions of neutrophils that phagocytosed the labeled bacteria were analyzed using BD FlowJo version 10.10.0 software.

The second of each duplicate was transferred into 24-well tissue culture plates, topped up with 1.4 ml of RPMI complete media containing 10% FBS, 1% sodium pyruvate, 1% GlutaMax, and 1% Non-essential amino-acids (all from Gibco), incubated at 37 °C humidified incubator with 5% CO_2_ for 18−22 hours and culture supernatants collected for DuoSet IL-8 sandwich ELIZA according the manufacturer’s instructions (R&D Systems, Catalog # DY208). The cell pellets were washed, fixed and flow cytometry data acquired as described above.

### Fluorescence microscopy of cytospin derived from flow cytometry samples

Approximately 50,000 leftover neutrophils from each sample analyzed by flow cytometry were centrifuged at 1200 RPM for 5 min at room temperature, the supernatant discarded, and cells resuspended in 200 μl PBS containing 1% BSA. The cell suspensions were then cytocentrifuged onto glass slides at 1000 RPM for 5 min. Slides were mounted with Prolong Diamond Antifade Mounting media containing DAPI (Invitrogen) and imaged at 100X using an Olympus BX51 fluorescence microscope operating the Cell Sense software. Both DAPI and green channels were captured, and images were merged using the Cell Sense software.

### Dual fluorescence and bright field live imaging

Neutrophils co-cultured with labeled bacteria for 24 h were harvested, washed, seeded into a flat bottom 96-well plate and live imaged under bright field and green fluorescence channels using an Olympus CK X41 inverted microscope. Bright field and fluorescence images generated from the same location were merged using Olympus Stream Essentials.

### Comparative genomics

Comparative genomics between *Sutterella* sp. KLE1602 and four other complete genomes classified to *S. wadsworthensis*_A in the Genome Taxonomy Database (GTDB),^22^ GC content calculation and phage mining was performed in Proksee.^23^ Visualization of the contig containing the IgA protease was performed in Proksee^23^ following annotation with Prokka.^24^ Blastn searches against the genes within the contig were performed in the NCBI database. Comparative genomics between *Sutterella* sp. KLE1602 and four metagenome-assembled genomes (MAGs) classified to *S. wadsworthensis*_A was also performed in Proksee.^23^ The four MAGs were obtained from a previously published dataset.^25^

### Metagenomic screen for IgA proteases

Metagenomic reads from samples from 30 countries were obtained from publicly available datasets (see Supplementary table 1 for accession numbers). Reads were screened for IgA protease homologs (both *S. wadsworthensis* and *Campylobacter concisus*) using DIAMOND v2.1.12 in BLASTX mode using default parameters. For each sample, the number of reads, hits to the database and alignment statistics (percentage identity, alignment length, and e-value) were recorded. Reads with >90% identity to *Sutterella* IgA protease for ≥20 nucleotides were consistently more similar to the protease at the same length than any other sequence when searched against NCBI. Thus, this cut-off was selected to assign positivity to the fastq file. Fastq files that only contained reads with >90% identity to *Sutterella* IgA protease for <20 nucleotides were manually validated for positivity through two methods: (1) reads were extracted and searched against NCBI nr database with any reads showing >50% identity to another species at greater length considered a false positive; (2) where available, paired sequencing samples were checked for robust evidence of positivity. In most cases these reads were assigned as false positives when manual validation was applied and samples were then considered negative for the protease.

To visualize read coverage, metagenomic reads with a 100% match identity to the gene encoding *S. wadsworthensis* IgA protease were first extracted using SeqKit (v2.5.1). Extracted reads were then aligned to the samples using BWA-MEM2 (v2.2.1) with default parameters. The resulting SAM file was converted to BAM format, sorted and indexed using SAMtools (v1.20) to produce the alignment file required for ggcoverage. Read depth across the gene encoding the IgA protease was computed and visualized in R using the ggcoverage (v1.4.1)^26^ and ggplot2 packages. Single-nucleotide resolution coverage was extracted for the entire gene region and coverage depth against the BAM file was plotted as a bar chart.

### Microbiome profiling

Metagenomic reads from a subset of the above samples corresponding to individuals from China and Fiji (Supplementary table 2) were analyzed. Raw sequencing reads belonging to the same sample were concatenated into a single set of forward and reverse fastq files and were then quality trimmed using the Read_qc module within MetaWRAP v1.3.2^27^ to trim adapters and remove human contamination with default parameters. Quality filtered reads were analyzed using MetaPhlAn4 (database vOct22)^28^ to generate tables with relative abundances of taxa classified to the species-genome bin (SGB) level using a marker-based analysis (t_ level in MetaPhlAn4 output). Alpha diversity measures Margalef’s richness, Pielou’s evenness and Shannon’s diversity index at the SGB level (t_) was calculated using Primer-e v6. SGB relative abundances were center-log transformed using the Tjazi package (clr_lite option with method set at logunif and 1000 replicates) in R v4.3.2. Square-root transformations, creation of resemblance matrices of Aitchinson distances (Euclidean distance on clr transformed data) or Bray-Curtis dissimilarities, principal component analysis (PCA), permutational multivariate ANOVA (PERMANOVA), and permutational analysis of dispersions (PERMDISP) were performed using Primer-e v6. All other statistical analysis as described, including testing for normal distribution using the Shapiro-Wilk test, were performed using GraphPad Prism v10. Per taxon analysis was performed using MaAsLin2,^29^ with settings of minimum abundance = 0, minimum prevalence = 0.1, normalization = TSS, transformation = log, analysis method = lm, and correction = BH.

### Sequence alignment and phylogeny

*C. concisus* IgA protease was identified through a blastn search using the NCBI database and the *S. wadsworthensis* IgA protease nucleotide sequence. Nucleotide sequence alignment among *S. wadsworthensis* IgA protease, *C. concisus* IgA protease and other IgA proteases was performed using Clustal Omega.^30^ The pairwise alignment between *S. wadsworthensis* IgA protease and *C. concisus* IgA protease was visualized using ESPript v3.^31^ For the multiple alignment, the tree file generated by Clustal Omega was extracted and visualized using Treeviewer.^32^

## Results

### Sutterella *sp KLE1602 possesses a human IgA protease*

Searches of the human IgA1 protease of *N. gonorrhoeae* against all available *Sutterella* proteomes in NCBI using PSI-BLAST revealed that the genome of *Sutterella* sp KLE1602 (GenBank: LTDB01000061.1) contained a coding sequence at position 10669 to 14724 that encoded a protein sequence corresponding to an IgA-specific serine endopeptidase (Uniprot: A0A139K5S6). The peptidase S6 domain for *Sutterella* sp KLE1602 putative IgA protease is from amino acid 32 to 285 while the peptidase domain of human IgA1 protease of *N. gonorrhoeae* is from amino acid 28 to 322. Alignment of the peptidase domains showed homology at the 5 amino acid residues (*Sutterella* sp KLE1602 _238_GDSGS_242_) corresponding to the active site serine of other serine proteases.^33^ Protease cleavage of the human IgA1 occurs in the 22-amino acid-hinge-region with glycosylated serine and threonine residues.^34^ Notably, *Sutterella* sp KLE1602 is classified as *S. wadsworthensis*_A in GTDB which is an independent species designation to *S. wadsworthensis*, the type species of the *Sutterella* genus. However, no other *S. wadsworthensis*_A representatives from GTDB (Supplementary table 3) possess a protein with homology to KLE1602 IgA protease.

**Figure 1.**
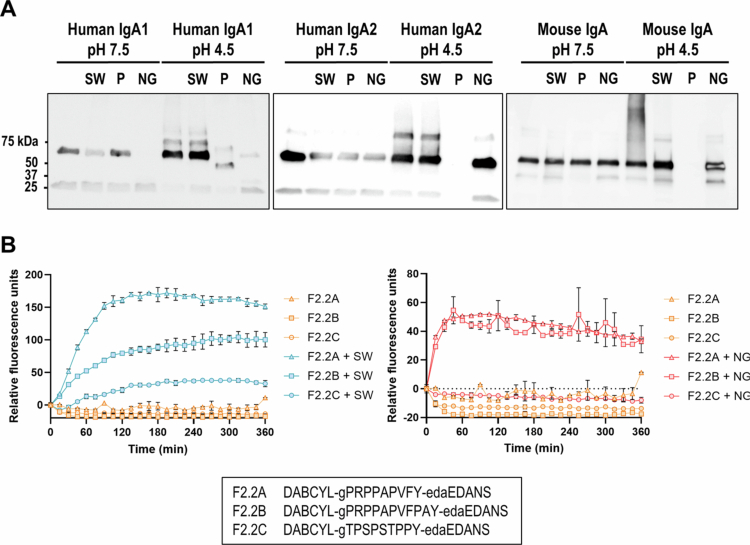
Activity of *S. wadsworthensis* IgA protease against human and mouse antibodies. A: Blots of human IgA1, human IgA2 and mouse IgA undigested or digested by *S. wadsworthensis* IgA protease (SW), pepsin (*P*) or *Neisseria gonorrhea* IgA protease (NG) at pH 7.5 or pH 4.5. B: Digestion of three human IgA1 mimics by *S. wadsworthensis* IgA protease (left) or *Neisseria gonorrhea* IgA protease (right) over time as measured by Förster resonance energy transfer (FRET). Peptides (F2.2A, F2.2B and F2.2C) are listed below. *n* = 3 per condition and errors are SEM. For *S. wadsworthensis*, F2.2A, F2.2B and F2.2C were not significantly different to each other while all remaining groups were different. For *Neisseria gonorrhea*, F2.2A + NG and F2.2B + NG were significantly different to all other groups. Comparisons were performed using one-way ANOVA with a post hoc Tukey’s multiple comparisons test.

We expressed and purified recombinant *Sutterella* sp KLE1602 IgA protease and established whether it could degrade human IgA1, human IgA2 and mouse IgA at pH 7.5 and 4.5. Capacity to degrade IgA was compared to pepsin and *N. gonorrhoeae* IgA1 protease. *Sutterella* sp KLE1602 IgA protease digested human IgA1 and IgA2 at pH 7.5 but did not possess activity at acidic pH (Figure 1A). It did not degrade mouse IgA at either pH (Figure 1A). *N. gonorrhoeae* IgA1 protease showed activity against human IgA1 at both pH conditions and activity against human IgA2 at pH 7.5 but no activity against mouse IgA (Figure 1A). Pepsin caused almost complete digestion of all three IgAs at pH 4.5 (Figure 1A).

We confirmed the results with FRET assays through monitoring IgA substrate digestion by *Sutterella* sp KLE1602 IgA protease and *N. gonorrhoeae* IgA1 protease. Three FRET peptides were used (Figure 1B), the F2.2 peptide (named F2.2A here) derived from Choudary et al.^35^ F2.2B a modified version of F2.2 that contained additional PA residues at the C-terminus to further test cleavage specificity, and F2.2C a peptide of 9 amino acid residues homologous to the human IgA1 hinge. *Sutterella* sp KLE1602 IgA protease cleaved all three peptides with different magnitude (Figure 1B left panel) whereas *N. gonorrhoeae* IgA1 protease cleaved F2.2A and F2.2B but not F2.2C (Figure 1B right panel).

*Sutterella* sp KLE1602 possesses a functional human IgA1 and IgA2 protease. The inability of *N. gonorrhoeae* IgA1 protease to digest F2.2C, while consistent to published results,^35^ revealed differing IgA digestion dynamics between the two proteases.

### Sutterella *IgA protease can modify infection outcomes in host cells*

**Figure 2.**
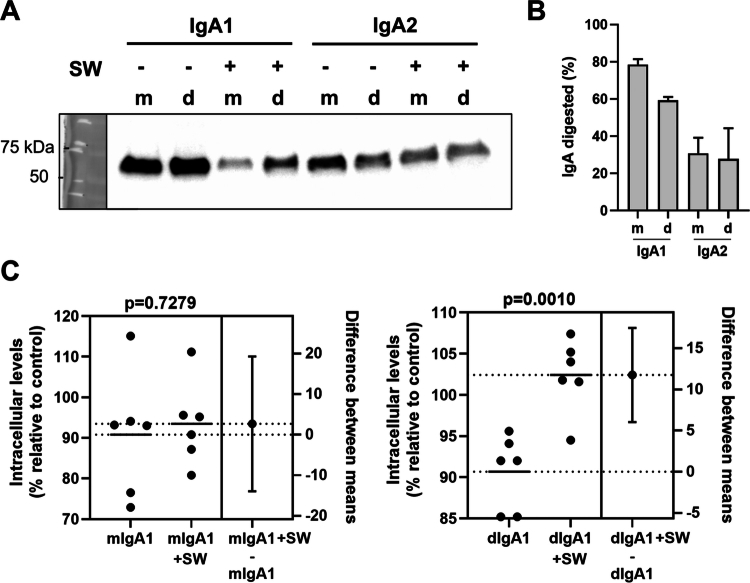
Functional effects of *S. wadsworthensis* IgA protease in a bacterial infection model. A: Activity of *S. wadsworthensis* IgA protease against human IgA1 and IgA2 specific to *Campylobacter jejuni*. Representative western blot showing degradation of monomeric (m) and dimeric (d) human IgA1 and IgA2 by *S. wadsworthensis* IgA protease (SW). B: Level of digestion of monomeric and dimeric human IgA1 and IgA2 by *S. wadsworthensis* IgA protease. Data is mean of *n* = 3 and errors are SEM. C: Effects of digestion of IgA by *S. wadsworthensis* IgA protease on intracellular levels of *C. jejuni* following infection of colonic epithelial cells. Relative intracellular levels of *C. jejuni* with undigested or digested monomeric or dimeric IgA1 were compared using unpaired t-test (Shapiro-Wilk test for normality: all *p* > 0.15; F-test for variance: all *p* > 0.42). Relative intracellular levels per condition were calculated by comparing intracellular levels of *C. jejuni* with undigested or digested IgA to those without IgA. *n* = 6 per group.

As recombinant *Sutterella* IgA protease did not digest mouse IgA in our hands, we could not perform *in vivo* mouse infection models. To establish functional relevance, we resorted to designing an infection model in human epithelial cells and an immune functional assay in primary human neutrophils. We required IgA antibodies that were specific to a particular human pathogen as well as the pathogen itself. Recombinant monoclonal human IgA antibodies (monomeric IgA1, dimeric IgA1, monomeric IgA2 and dimeric IgA2) against *C. jejuni* flagellar capping protein FliD were generated.^20^ *Sutterella* sp KLE1602 IgA protease could degrade all versions of the recombinant IgA antibodies with differing efficiencies (Figure 2A,B), with the monomeric IgA1 being degraded the most and dimeric IgA2 degraded the least (Figure 2B).

**Figure 3.**
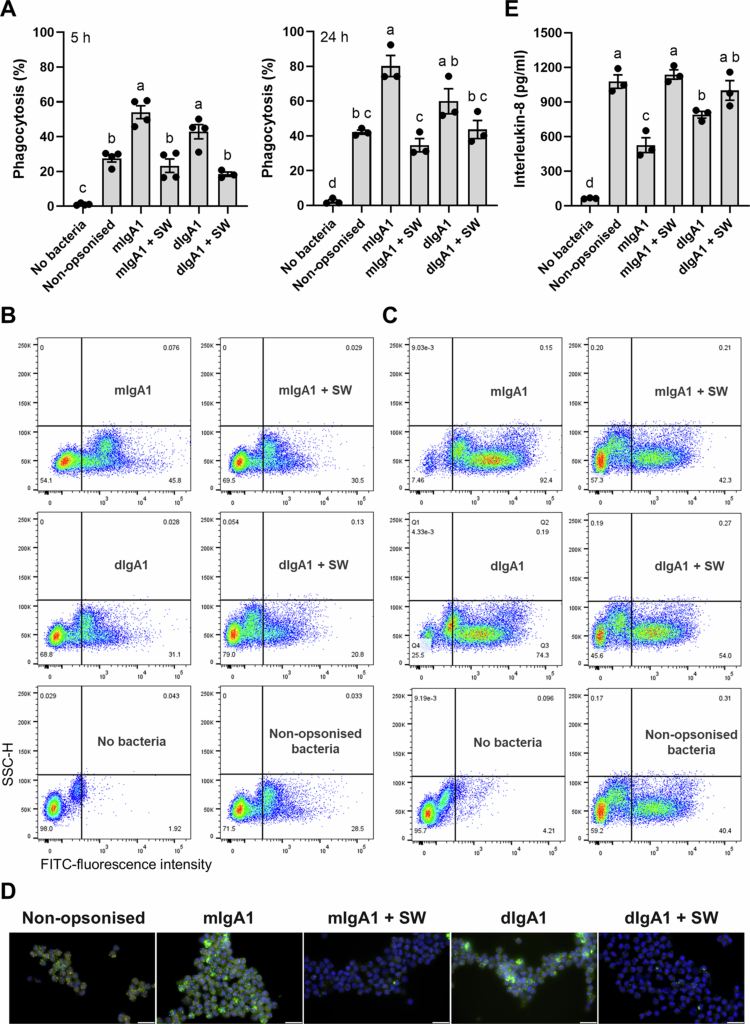
Functional effects of *S. wadsworthensis* IgA protease on phagocytosis by primary human neutrophils. A: Proportion of neutrophils that phagocytosed fluorescein isothiocyanate (FITC)-conjugated *C. jejuni* at 5 h (left) and 24 h (right) as detected by flow cytometry. Conditions tested include no bacteria, bacteria without antibody (non-opsonised), bacteria with undigested monomeric IgA1 (mIgA1), bacteria with monomeric IgA1 digested with *S. wadsworthensis* IgA protease (mIgA1 + SW), bacteria with undigested dimeric IgA1 (dIgA1), bacteria with dimeric IgA1 digested with *S. wadsworthensis* IgA protease (dIgA1 + SW). Comparisons for 5 h (Shapiro-Wilk test for normality: all *p* > 0.0758; Brown-Forsythe test for variance: *p* = 0.0715) and 24 h (Shapiro-Wilk test for normality: all *p* > 0.0699 except mIgA1 *p* = 0.0091; Brown-Forsythe test for variance: *p* = 0.8532) were performed using one-way ANOVA with a post hoc Tukey’s multiple comparisons test. Statistical differences are presented as compact letter display. *n* = 3-4 for all groups. B: Representative flow cytometry plots of phagocytosis of FITC-conjugated bacteria by neutrophils for each condition at 5 h timepoint. C: Representative flow cytometry plots of phagocytosis of FITC-conjugated bacteria by neutrophils for each condition at 24 h timepoint. Y-axis, side scatter height (SSC-H); X-axis, fluorescence intensity of neutrophils that ingested FITC-conjugated bacteria (+ve phagocytosis). D: Representative fluorescent microscopy images of cytospins for each condition. Bacteria are labeled in green with a DAPI nuclear counterstain. E: Levels of secreted interleukin-8 at 24 h (top right corner). Comparisons were performed using one-way ANOVA with a post hoc Tukey’s multiple comparisons test (Shapiro-Wilk test for normality: all *p* > 0.3768; Brown-Forsythe test for variance: *p* = 0.5814). Statistical differences are presented as compact letter display. *n* = 3 for all groups.

We performed gentamicin protection assays using *C. jejuni* 81116 and the colonic epithelial cell-line HT-29 where the bacterium was incubated with undigested or digested monomeric or dimeric IgA1 (Figure 2C). Despite the monomeric IgA1 being degraded to a higher efficiency than the dimeric IgA1, we only observed a significant increase in intracellular levels of the pathogen relative to control when the dimeric IgA1 was used (Figure 2C). Antibody digestion appeared to recover the ~10% loss in intracellular levels of *C. jejuni* on incubation with dimeric IgA1 (Figure 2C). We then examined the effects of *Sutterella* sp KLE1602 IgA protease on phagocytic activity of primary human neutrophils at two timepoints. Neutrophils were incubated with *C. jejuni* 81116 opsonised with undigested or digested monomeric or dimeric IgA1 for 5 or 24 h (Figure 3A). Digestion of human IgA1 had a consistent impact on the ability of neutrophils to phagocytose *C. jejuni*, lowering levels to that of non-opsonised bacteria (Figure 3A−D; Supplementary figure 1). The statistical significance of the effect was lost for the dimeric IgA1 at 24 h (Figure 3A right panel), possibly due to an increase in intracellular levels of non-opsonised *C. jejuni*. The impact on phagocytosis efficiency also corresponded with secreted IL-8 levels which were higher when the recombinant antibodies were digested and reached levels measured for non-opsonised *C. jejuni* (Figure 3E).

*Sutterella* sp KLE1602 IgA protease can modify dynamics of infection in human cells *in vitro*, with the impact appearing to be more potent in primary immune cells than epithelial cells.

### Comparative genomics indicates that Sutterella IgA protease was located on a unique contig and had similarity to another gastrointestinal organism

**Figure 4.**
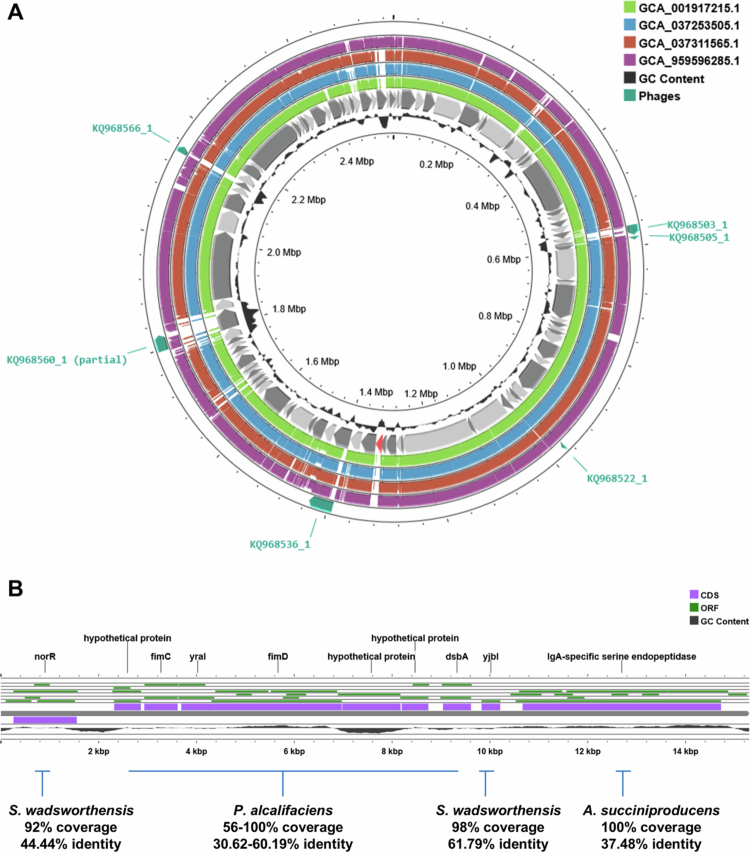
Genomic analysis of *S. wadsworthensis* IgA protease. A: Alignment of *S. wadsworthensis* KLE1602 genome (internal gray) against four other strains classified as *S. wadsworthensis*_A with complete genomes (100%; green, blue, red and purple). KLE1602 is classified as *S. wadsworthensis*_A in GTDB. Contig containing IgA protease is highlighted in red and is absent in the other four strains. Contigs predicted to contain phage-associated regions are highlighted in teal arrows (outer circle). B: Visual representation of contig containing *S. wadsworthensis* IgA protease. Predicted open reading frames (ORFs; green), annotations (Prokka; purple) and GC content (black) are presented. Region containing putative fimbrial-associated molecules with sequence similarity to *Providencia alcalifaciens* is flanked by two genes with similarity to genes within other *S. wadsworthensis* genes. The gene encoding IgA protease shows no homology to any genes within other available *S. wadsworthensis* genomes but exhibits similarity to a gene within *Anaerobiospirillum succiniciproducens*.

We assessed how *Sutterella* sp KLE1602 may have gained the IgA protease activity. We aligned the *Sutterella* sp KLE1602 genome with the four representatives of *S. wadsworthesis*_A that had 100% complete genomes (GTDB, Figure 4A). We established that the IgA protease was present on a unique contig that was absent in the other four genomes. We confirmed this by aligning the KLE1602 genome against 5 high-quality metagenome-assembled genomes classified to *S. wadsworthesis*_A from an independent study^25^ (Supplementary figure 2). Phage analysis of KLE1602 predicted that the contig was not derived from a phage integration event (Figure 4A). Genomic analysis of the contig containing the IgA protease (contig: KQ968533.1) suggested that one region had high similarity to *Providencia alcalifaciens* (flanked by genes conserved in some *S. wadsworthensis* genomes) whereas the IgA protease was an independent region of the contig with similarity to the gastrointestinal organism *Anaerobiospirillum succiniciproducens* (Figure 4B), showing substantially higher sequence coverage and identity (%) than to other well-studied IgA proteases.

*Sutterella* sp KLE1602 IgA protease does not appear to have originated from a phage integration event but showed similarity to an IgA protease from another mammalian gastrointestinal organism.

### Sutterella IgA protease can be detected in global human metagenomes

**Figure 5.**
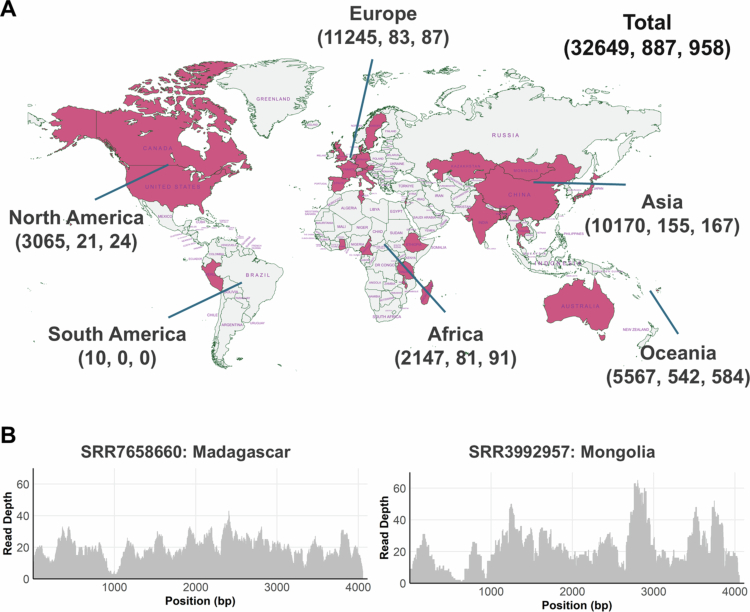
Analysis of global prevalence of *S. wadsworthensis* IgA protease in metagenomic data. A: Map of fecal shotgun metagenomic sequencing data screened for the presence of *S. wadsworthensis* IgA protease. Countries (*n* = 30) where data was analyzed are highlighted in red. A total of 32649 fastq files were screened, with 958 found to contain reads aligning to IgA protease at >90% of which 887 contained reads that aligned at 100% similarity. Total fastq files, detection at 100% similarity, and detection at >90% similarity are presented per global region. Some fastq files did not have metadata linking to location. Created with MapChart. B: Read coverage of gene encoding *S. wadsworthesis* IgA protease in two metagenomic samples from distinct regions (Madagascar and Mongolia). Two samples with moderate to high coverage were selected and only coverage from R1 fastq file was included.

Given the rare presence of the IgA protease sequence within *Sutterella* proteomes, we aimed to establish the epidemiological relevance of this activity in the human gut microbiome. A total of 32,649 non-redundant fastq files from control individuals from 30 countries were downloaded and searched (Supplementary tables 1,4). These corresponded to sequencing 17,698 individual samples based on paired- and single-end information. When considering detection at >90% identity as a positive detection, we observed a presence in fastq files ranging from 0.78% and 0.77% in North America and Europe to 1.64% and 4.24% in Asia and Africa, respectively (Figure 5A). The prevalence in Oceania was substantially higher at 10.49% (Figure 5A); however, this was a consequence of high sampling and high detection specifically from Fiji as we did not detect *Sutterella* IgA protease in any healthy Australian individuals profiled. While no detection was observed in South America, this population was not sampled appropriately to make any conclusions. To confirm that detection of *Sutterella* IgA protease in metagenomes was robust and not a consequence of random sequence similarity at a specific site in the protease, we selected two samples with moderate to high coverage from distinct cohorts and countries (Madagascar and Mongolia). We assessed the coverage of reads found to have 100% identity to the protease and observed good coverage across the full protease nucleotide sequence (Figure 5B). We also extracted reads with a similarity to the protease between 90-91% and performed blastx searches against the NCBI database (Supplementary table 5). All reads at that similarity % for >20 nucleotides were robustly identified as *Sutterella* KLE1602 IgA protease. We reassessed evidence of positivity across all samples and identified 61 fastq files that were assigned as positive based on weak evidence (read coverage < 20 nucleotides with no additional reads similar to the protease). These fastq files were then manually validated, with 6 showing clear similarity to the protease while 55 assigned as false positives (Supplementary table 1).

*Sutterella* IgA protease is present in the human gut microbiome globally, showing differing rates of prevalence across regions, with a relatively high detection rate in Africa and an even higher rate in Fiji.

### Presence of sutterella IgA protease is associated with differences in the human gut microbiome

**Figure 6.**
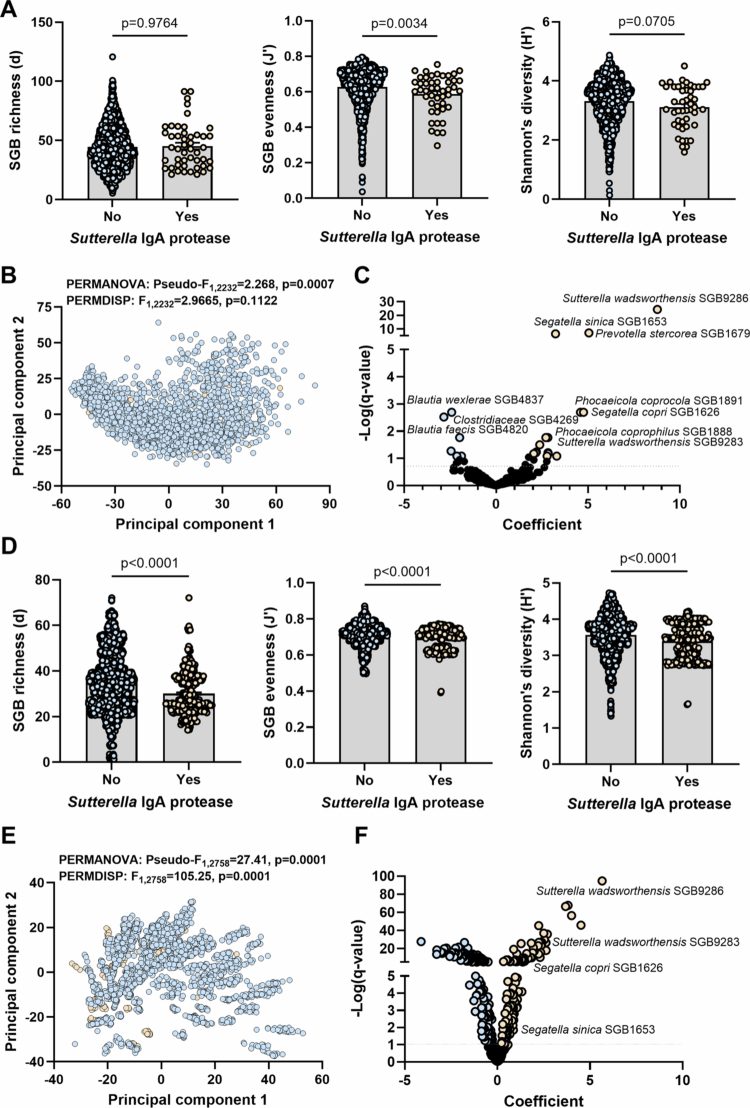
Association between *Sutterella* IgA protease positivity and gut microbiome profile. 2234 and 2760 human gut metagenomes from China and Fiji, respectively were analyzed using MetaPhlAn v4 to obtain taxonomic profiles at the t_ classification for species genome bins (SGB). A: Comparisons of alpha diversity metrics (Margalef’s richness, Pielou’s evenness and Shannon’s diversity index) between samples from China positive for the IgA protease (containing reads with >90% identity to protease) and those that were negative. Statistical comparisons were performed using Mann-Whitney tests following confirmation of lack of normal distributions using Shapiro-Wilk test. B: Ordination using principal component analysis of Aitchinson distances on samples from China. Statistical comparisons were performed using PERMANOVA and PERMDISP. Results were confirmed using Bray-Curtis dissimilarities calculated on square-root transformed relative abundances (PERMANOVA: Pseudo-F_1,2232_: 2.6506, *p* = 0.0001; PERMDISP: F_1,2232_: 1.5115, *p* = 0.2873). C: Per taxon analysis on samples from China represented as a volcano plot of MaAsLin2 coefficients and q-values, with taxa found to have q < 0.1 being highlighted in orange (higher in positive) and blue (lower in positive). Taxa with q < 0.02 are labeled. D: Comparisons of alpha diversity metrics (Margalef’s richness, Pielou’s evenness and Shannon’s diversity index) between samples from Fiji positive for the IgA protease (containing reads with > 90% identity to protease) and those that were negative. Statistical comparisons were performed using Mann-Whitney tests following confirmation of lack of normal distributions using Shapiro-Wilk test. E: Ordination using principal component analysis of Aitchinson distances on samples from Fiji. Statistical comparisons were performed using PERMANOVA and PERMDISP. Results were confirmed using Bray-Curtis dissimilarities calculated on square-root transformed relative abundances (PERMANOVA: Pseudo-F_1,2758_: 34.217, *p* = 0.0001; PERMDISP: F_1,2758_: 65.809, *p* = 0.0001). F: Per taxon analysis on samples from Fiji represented as a volcano plot of MaAsLin2 coefficients and q-values, with taxa found to have q < 0.1 being highlighted in orange (higher in positive) and blue (lower in positive). Only relevant taxa are labeled.

We explored if the presence of *Sutterella* IgA protease within a metagenome was associated with differences in microbiota diversity. To investigate this, we analyzed 2234 independent human gut metagenomes from China (Supplementary table 2) using MetaPhlAn v4 and obtained taxonomic profiles at the t_ classification (SGB references set at 95% Average Nucleotide Identity). We stratified samples based on IgA protease positivity (positive detection set at >90% identity against the protease) and assessed if there were differences in microbiota alpha or beta diversity. We chose the Chinese cohort because we posited that analysis of the global cohort would obscure any potential relationships due to microbiome variation across geography and that the Chinese cohort would be optimal as it represented a large relatively homogeneous cohort compared to other sampled populations, with a moderate level of protease positivity (1.84%, *n* = 41/2234 positive). We observed a significant difference in SGB evenness, with a lower evenness found in positive samples (*p* = 0.0034; Figure 6A). Shannon’s diversity was also lower; however, this comparison did not reach significance (*p* = 0.0705; Figure 6A). In contrast, SGB richness was not different between the groups (*p* = 0.9764; Figure 6A). Beta-diversity was also significantly different between the two groups (Figure 6B) regardless of the dissimilarity or distance calculated (Aitchinson distance: *p* = 0.0007; Bray-Curtis dissimilarity: *p* = 0.0001), with a notable lack of significance in homogeneity of dispersions (Aitchinson distance: *p* = 0.1122; Bray-Curtis dissimilarity: *p* = 0.2873) indicating the PERMANOVA represents a true shift in composition not dispersion. Consistently, we identified 22 SGBs to be significantly differentially abundant (q < 0.1) when positive samples were compared to negative ones (33 SGBs at q < 0.25) (Supplementary table 6). These included two *S. wadsworthensis* SGBs enriched in positive samples (Figure 6C), validating the stratification. Further, two SGBs classified to the dominant genus *Segatella* were among those enriched in positive samples, which could explain why positive samples also had lower evenness.

We then validated these results by analyzing an independent cohort from Fiji (*N* = 2760 metagenomes; *n* = 291 samples positive at >90% identity against the protease). We again observed lower SGB evenness and Shannon’s diversity in positive samples (*p* < 0.0001; Figure 6D). However, in this cohort, SGB richness was also significantly lower in positive samples (*p* < 0.0001; Figure 6D). Beta-diversity was significantly different between the two groups (Figure 6E) regardless of the dissimilarity or distance calculated (Aitchinson distance: *p* = 0.0001; Bray-Curtis dissimilarity: *p* = 0.0001), but this should be taken with caution as there was a significant difference in the homogeneity of dispersions (Aitchinson distance: *p* = 0.0001; Bray-Curtis dissimilarity: *p* = 0.0001). In this cohort, 329 SGBs were found to be significantly differentially abundant (q < 0.1) when positive samples were compared to negative ones (309 SGBs at q < 0.05) (Supplementary table 7). These included the same two *S. wadsworthensis* SGBs (SGB9286 and SGB9283) enriched in positive samples (Figure 6F). Moreover, the same two SGBs classified to the dominant genus *Segatella* were among those enriched in positive samples (Figure 6F).

The prevalence of the two *S. wadsworthensis* SGBs found to be significantly enriched in positive samples were *n* = 467 of 2234 (20.9%) and *n* = 984 of 2760 (35.6%) for SGB9286 in China and Fiji, respectively and *n* = 466 of 2234 (20.8%) and *n* = 741 of 2760 (26.8%) for SGB9283 in China and Fiji, respectively (Supplementary tables 6, 7). While several SGBs that classified to *Sutterella* were detected, SGB9286 and SGB9283 were the only SGBs classified to *S. wadsworthensis* that were detected in the two cohorts.

Gut microbiome samples positive for the *Sutterella* IgA protease have a significantly different profile to those that are negative. This would suggest that either the presence of the protease may shape the host’s gut microbiome profile, certain microbiome profiles such as those rich in *Segatella* provide an optimal niche for IgA protease^+^ *Sutterella wadsworthensis* colonization, or there exists a correlated environmental factor that modifies the microbiome towards *Segatella* enrichment and colonization by IgA protease^+^ *Sutterella wadsworthensis*.

### Homology searches against Sutterella IgA protease identifies novel IgA proteases from clinically relevant members of the human microbiome

**Figure 7.**
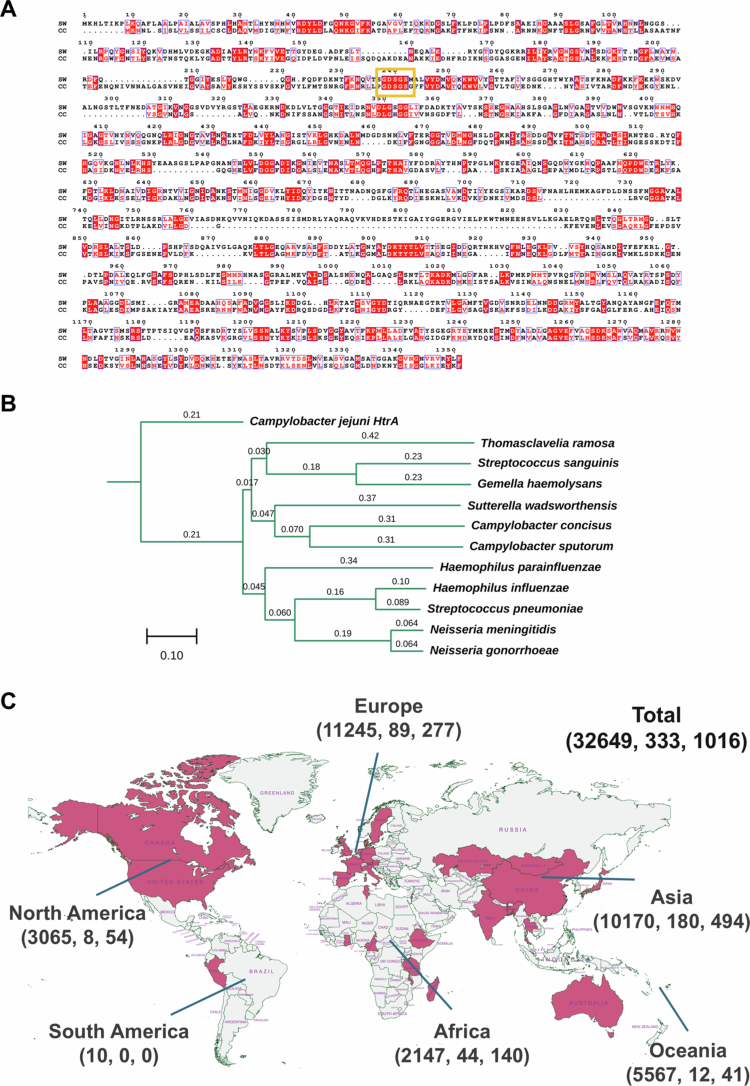
Homology searches against *S. wadsworthensis* IgA protease enable detection of novel bacteria containing IgA proteases in human gut microbiome. A: Sequence alignment between *S. wadsworthensis* IgA protease and putative *Campylobacter concisus* IgA protease. Alignment was performed using Clustal Omega and active serine region is highlighted with a yellow box. B: Phylogenetic analysis of protein sequences corresponding to known or putative IgA proteases from different bacterial species. Numbers above branches are length as outputted by Clustal Omega. Outgroup is *C. jejuni* secreted serine protease HtrA. Well-characterized IgA proteases from pathogens cluster separately from those from known members of the oral and gut microbiotas. C: Map of fecal shotgun metagenomic sequencing data screened for the presence of *C. concisus* IgA protease. Countries (*n* = 30) where data was analyzed are highlighted in red. A total of 32649 fastq files were screened, with 1016 found to contain reads aligning to IgA protease at >90% of which 333 contained reads that aligned at 100% similarity. Total fastq files, detection at 100% similarity, and detection at >90% similarity are presented per global region. Some fastq files did not have metadata linking to location. Created with MapChart.

Given the sequence divergence of the *Sutterella* IgA protease from other well-studied pathogen IgA1 proteases, we explored if searches against the *Sutterella* IgA protease would reveal novel IgA proteases within members of the human microbiota. Homology searches using blastp showed that clinically important members of the human oral and gut microbiotas, emerging *Campylobacter* spp. possessed proteins with a reasonable level of coverage and identity (e.g., *C. concisus* 98% coverage and 23.48% identity), a predicted peptidase S6 domain, as well as a conserved active site serine (GDSGS) (Figure 7A). Phylogenetic analysis of IgA protease amino acid sequences from pathogens and members of the microbiota such as the recently identified *Thomasclavelia ramosa* IgA protease indicated distinct clustering between the two groups of organisms, where the *Sutterella* and *Campylobacter* proteins clustered closely together (Figure 7B). We also established the epidemiological relevance of *Campylobacter* IgA protease in the same global metagenome dataset as above (Figure 7C), and when considering a strict cut-off of detection at >90% identity as a positive, we observed higher prevalence rates than *Sutterella* IgA protease across most regions sampled appropriately except for Oceania (i.e., Fiji) where there was low prevalence (0.74%). Notably, we observed a larger difference between prevalence at 100% identity and >90% identity for *Campylobacter* when compared to *Sutterella* (Figure 5A, Figure 7C); this potentially reflects the uniqueness of *Sutterella* IgA protease as opposed to the presence of *Campylobacter* IgA protease across multiple *Campylobacter* species leading to more true detections at lower sequence homology.

Searches against *Sutterella* IgA protease reveal a novel group of IgA proteases within members of the human oral and gut microbiotas, and these proteins can also be detected in human gut metagenomes from various countries.

## Discussion

*Sutterella wadsworthensis* is an anaerobic, bile-resistant bacterium that was initially isolated from appendiceal and peritoneal fluid specimens^36^ but later found to be a common member of the human gut microbiota^12^ that is more consistently detected in gut mucosal samples than fecal samples.^18^ Its clinical relevance in the gut remains contentious having been previously implicated in the pathogenesis of IBD,^37^^,^^38^ findings that were not replicated in other studies.^19^^,^^39^ It has been associated with treatment failure in UC^13-17^ despite it not inducing epithelial inflammation or altering monolayer integrity.^18^^,^^19^ Here, we find that a subset of *S. wadsworthensis* strains classified to *S. wadsworthensis*_A encode an IgA protease that is capable of degrading human IgA1 and IgA2 but not mouse IgA. Exposure to the IgA protease impacts intracellular levels of bacteria in models of epithelial and immune cell infection with *C. jejuni* that employ a unique *C. jejuni*-specific human recombinant antibody. Genomic analysis suggested *S. wadsworthensis* acquisition of the IgA protease through horizontal gene transfer rather than phage integration, with the closest homolog being from a bacterium commonly found in the gut microbiota of dogs and cats. Metagenomic analysis of data from 30 countries indicated that this protease can be detected in the gut microbiome of individuals globally, and that its presence in the microbiome is associated with lower species evenness, a difference in composition driven in part by enrichment of *Segatella* spp. Homology searches and phylogenetic analysis using the *S. wadsworthensis* IgA protease identified putative IgA proteases from another clinically relevant taxon, namely *Campylobacter* spp. and that these microbiota-derived IgA proteases clustered separately from pathogen-derived IgA proteases.

We have reported that *S. wadsworthensis* was enriched in patients with UC that had negative clinical outcomes following fecal microbiota transplantation (FMT), and that this bacterium was a marker of ineffective donor batches.^14^^,^^15^ These findings were supported by a study assessing the additive effect of pectin on FMT efficacy in UC, where patients receiving pectin and FMT had lower disease activity scores and less *Sutterella* than patients on FMT alone.^17^ Moreover, in addition to microbiome-based interventions, pediatric patients with UC on standard care were more likely to have corticosteroid-free remission at one-year follow-up with a decrease in *Sutterella.*^13^^,^^16^ It would be of interest to explore if the IgA protease plays a direct role in therapeutic outcomes in patients with UC given the consistent findings for a role for *S. wadsworthensis*. Our findings also show that individuals with the IgA protease have a different microbiota profile. While it is unclear if the microbiota differences are cause or consequence, it is plausible that they may be involved in the outcomes of therapy. This is particularly relevant when considering that another marker of treatment failure following FMT in our clinical trial was enrichment of *Segatella copri,*^15^ a taxon found to be enriched in IgA protease positive individuals in the current study.

It has also been previously reported that the presence of *Sutterella* within the gut microbiota of mice contributed to a phenotype characterized by low fecal IgA levels.^40^ The authors observed that this low IgA phenotype was vertically transmissible from mum to offspring, laterally transferable through FMT or co-housing, as well as dominant given that co-housing of high and low IgA mice resulted in mice with low IgA rather than intermediate levels.^40^ Live *Sutterella* as well as their lysates were found to degrade secretory component of IgA, and mice with low IgA showed increased distal colon ulceration upon exposure to chemically induced injury when compared to their high IgA counterparts.^40^ While these findings would support a role for the IgA protease in colitis development, we did not observe digestion of mouse IgA when using the recombinant *S. wadsworthensis* IgA protease. It would be of interest to determine if there remains another IgA protease within *Sutterella* capable of degrading mouse IgA or if this protease can degrade mouse IgA under conditions not tested in our study, given that it can degrade human IgA2 with a shorter hinge region than IgA1.

Our study highlights the importance of strain-level resolution in microbiota studies. Current methods to analyze the microbiota, even when examining deep metagenome sequencing data, appear to struggle to differentiate between *S. wadsworthensis* that can produce IgA protease and those that do not, and identification of positive samples required specific sequence similarity searches of the protease against the metagenomes. Given the global prevalence of this subtype of *S. wadsworthensis*_A, the consequences of grouping the prevalent and relatively abundant IgA protease-negative *S. wadsworthensis* with that which can produce IgA protease on the interpretation of the role of the bacterium in human health may be far-reaching. This can also be seen in other contexts related to IgA, where different strains of the commensal *Bacteroides ovatus* have been shown to induce different levels of IgA in the gut.^41^

The complete contig containing the *S. wadsworthensis* IgA protease was absent in other *S. wadsworthensis*_A genomes studied. While a portion of the contig appeared to be derived from *P. alcalifaciens*, the IgA protease itself was most closely related to a putative IgA protease from *A. succiniciproducens*. This bacterium is a common member of the gut microbiota of dogs and cats,^42^ with animals considered a potential source of human infection by *A. succiniciproducens.*^43^ It can be speculated that interaction of the two bacterial species could have occurred during human infection, or alternatively within the gut microbiome of dogs and cats where both *A. succiniciproducens* and *S. wadsworthensis* can occur.^44^^,^^45^ It is worth noting that it is reported that dogs have a single subclass of IgA with a long hinge region similar to human IgA1,^46^ making them susceptible to protease cleavage. However, allelic variation has been shown to produce dog IgA with shorter hinges as well.^46^ In contrast, little is known about cat IgA but available *Felis catus* IgA constant region sequences in NCBI share more similarity to human IgA2 than IgA1 and specifically lack the long hinge of IgA1 on sequence alignment.

Another finding in our study was the identification of putative IgA proteases within *Campylobacter* spp. that inhabit the oral and gut microbiotas of humans. These *Campylobacter* spp. have also been implicated in the pathogenesis of IBD.^47^ However, *S. wadsworthensis*, when present, is substantially more abundant in the human gut environment than *Campylobacter* spp., increasing the likelihood of a consequential effect on the host. Nonetheless, the shared activity on the host immune system, the shared enigmatic role in human gut health and possible inflammaphilic nature warrants further investigation. Notably, when assessed as a collective, these microbiota-derived IgA proteases clustered separately from their pathogen-derived counterparts. This is most clearly highlighted in the case of IgA protease from *Streptococcus sanguinis*, a common member of the human oral microbiota, being particularly divergent from the IgA protease from *Streptococcus pneumoniae*, an inhabitant of the respiratory tract that can cause severe illness.^48^ Further investigation of the split between these two types of IgA proteases may shed light on their origins and impacts on human health.

This study has limitations. Despite *Sutterella* IgA protease not being able to digest mouse IgA, it would be important to validate the effects of this protease *in vivo*. While beyond the scope of the currently study, it would be plausible to orally administer digested and undigested recombinant *C. jejuni*-specific human IgA in the context of a mouse model of *C. jejuni* infection and investigate the differential impact on infection outcome and gut microbiota diversity. Moreover, it would also be important to obtain additional genomic evidence, particularly in the context of establishing the origin of this protease. Of interest would be to determine if *Sutterella* IgA protease could cleave the hinge region of dog and cat IgAs. Our prevalence analysis is based on similarity searches, and we opted for strict cut-offs of 90% and 100%. Despite applying manual checks and filters, we cannot completely rule out further false positives and the strict cut-offs may have led to false negatives as reads with 80-90% identity to the protease for a reasonable length were identified in the dataset.

In conclusion, we identify a functional human IgA protease in a previously assumed commensal bacterium and that this protease can modify the outcomes of infection by a globally relevant bacterial pathogen. Future work should explore the pervasiveness of IgA proteases within the microbiota and their clinical impact in the gut and other highly relevant environments such as the respiratory tract.

## Supplementary Material

Supplementary figures IgApro.docx Supplementary figures IgApro.docx

Supplementary tables IgAPro Rev.xlsx Supplementary tables IgAPro Rev.xlsx

## Data Availability

Shotgun metagenomic sequencing samples analyzed in this study are all publicly available and accession numbers are listed in the supplementary tables.
