# Supplementary material for: Immunoglobulin A protease from Sutterella wadsworthensis modifies outcome of infection with Campylobacter jejuni and is associated with microbiome diversity
Source: Gut Microbes. 2026 Jan 6;18(1):2611543. doi: 10.1080/19490976.2025.2611543 (PMC12785231; doi:10.1080/19490976.2025.2611543)

**Supplementary figures**

**Supplementary figure 1**

**Live microscopy images of primary neutrophils co-cultured with labelled *C. jejuni* for 24 h.** Images are merges from bright field and fluorescence microscopy. Conditions imaged include bacteria with undigested monomeric IgA1 (mIgA1), bacteria with monomeric IgA1 digested with *S. wadsworthensis* IgA protease (mIgA1 + SW), bacteria with undigested dimeric IgA1 (dIgA1), bacteria with dimeric IgA1 digested with *S. wadsworthensis* IgA protease (dIgA1 + SW), and bacteria without antibody (non-opsonised). Scale bar, 100 μm.


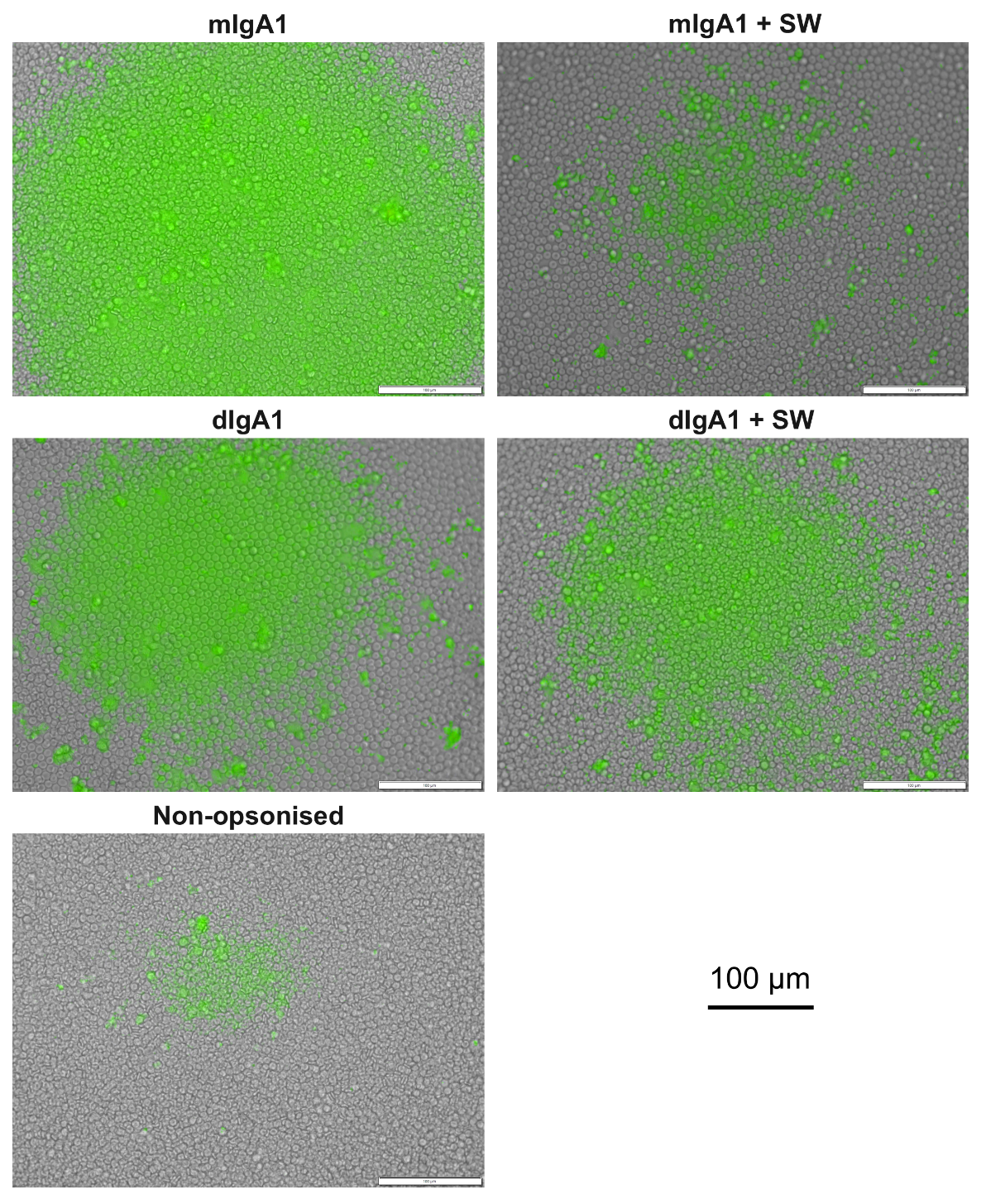


**Supplementary figure 2**

**Genomic analysis of *S. wadsworthensis* KLE1602.** Alignment of *S. wadsworthensis* KLE1602 genome (internal gray) against five near complete metagenome-assembled genomes (MAGs) classified as *S. wadsworthensis*_A (green, orange, blue, purple and brown). KLE1602 is classified as *S. wadsworthensis*_A in GTDB. Contig containing IgA protease is highlighted in red and is absent in the five other MAGs.


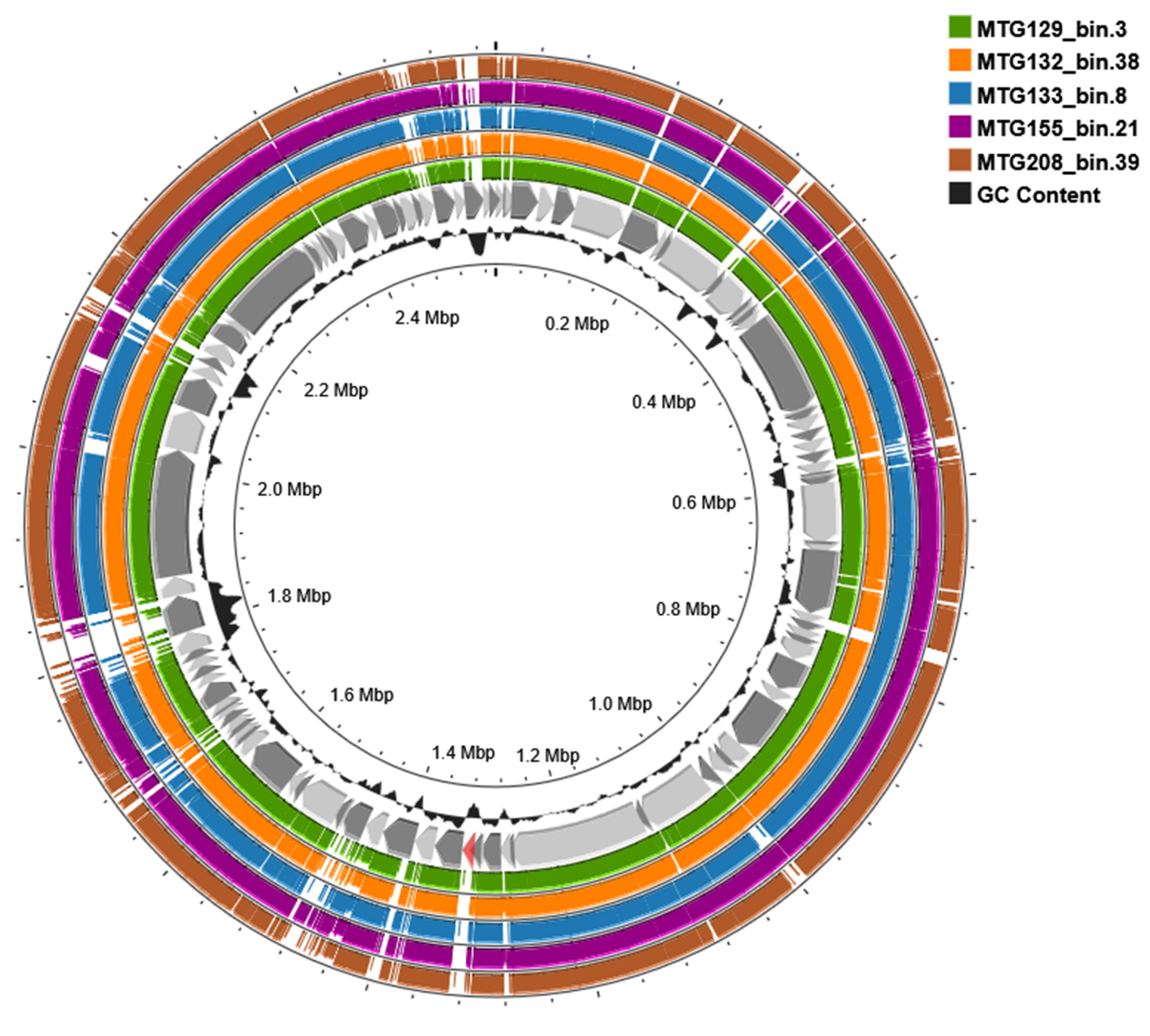

Supplement: Supplementary figures IgApro.docx [file KGMI_A_2611543_SM3476.docx]
